# Coastal Nurseries and Their Importance for Conservation of Sea Kraits

**DOI:** 10.1371/journal.pone.0090246

**Published:** 2014-03-26

**Authors:** Xavier Bonnet, François Brischoux, Christophe Bonnet, Patrice Plichon, Thomas Fauvel

**Affiliations:** 1 Centre d’Etudes Biologiques de Chizé, Unité Mixte de Recherche 7372 Centre National de la Recherche Scientifique et Université de La Rochelle, Villiers-en-Bois, France; 2 Centre Hospitalier Territorial Gaston Bourret, Nouméa, Nouvelle Calédonie, France; 3 Direction de l’Environnement, Nouméa, Province Sud, Nouvelle Calédonie, France; 4 Université Pierre et Marie Curie, Paris, France; Institut Pluridisciplinaire Hubert Curien, France

## Abstract

Destruction and pollution of coral reefs threaten these marine biodiversity hot stops which shelter more than two thirds of sea snake species. Notably, in many coral reef ecosystems of the Western Pacific Ocean, large populations of sea kraits (amphibious sea snakes) have drastically declined during the past three decades. Protecting remaining healthy populations is thus essential. In New Caledonia, coral reefs shelter numerous sea krait colonies spread throughout an immense lagoon (24,000 km^2^). Sea kraits feed on coral fish but lay their eggs on land. However, ecological information on reproduction and juveniles is extremely fragmentary, precluding protection of key habitats for reproduction. Our 10 years mark recapture study on Yellow sea kraits (*L. saintgironsi* >8,700 individuals marked) revealed that most neonates aggregate in highly localized coastal sites, where they feed and grow during several months before dispersal. Hundreds of females emigrate seasonally from remote populations (>50 km away) to lay their eggs in these coastal nurseries, and then return home. Protecting these nurseries is a priority to maintain recruitment rate, and to retain sea krait populations in the future.

## Introduction

The first global assessment of the conservation status of reptiles revealed that at least 20% of the species are under risk of extinction [1]. This study pointed out the need to increase research effort on tropical areas that are subjected to drastic habitat loss, and notably on snakes in which population information is deficient. The authors also recommended orienting conservation actions to alleviate the effects of habitat loss and harvesting. Indeed, snake populations are rapidly declining worldwide [2]–[12]. Although habitat loss and global changes are important factors for these declines [13], direct destruction of snakes also exert strong pressures on populations. Millions of individuals are killed worldwide every year, and hundreds of thousands are collected for leather and pet trades [8], [14], [15]. Unfortunately, conservation efforts devoted to this taxon remain modest [1].

Sea snakes are especially at risk [10]–[12], [16]–[17]. Very large numbers have been killed for food, skin industry, or as fishery bycatch [18]–[21]. Further, coral reefs that shelter most sea snake species (66 among 86 identified species, 77% [22]) represent the most severely devastated biome of the planet and more than half of the remaining reefs are under risk of collapse [23]. Overall, sea snakes living in coral reefs deserve urgent attention.

Sea kraits (Elapid snakes; *Laticauda* genus) are amphibious snakes widely distributed in the coral reefs of the East Indian and West Pacific Ocean [24]. They forage at sea but return on land, usually coralline islets, for resting, shedding skin, and digesting prey [25]–[26]. This dependence for coastal terrestrial habitats entails specific risks because rapid increase of human demography, rapid industrial and mining developments threaten coastlines [27]. In addition, these anthropogenic activities also entail a marked contamination of the prey consumed by sea snakes [28]. Many populations of sea kraits collapsed during the last three decades, notably due to habitat loss and massive harvesting for the leather industry [16], [18], [22]. Sea kraits are oviparous, and they lay their eggs on land; therefore, sea kraits are also vulnerable on land [24]–[25]. Communal nesting in tidal caves has been observed in two species (*L. semifasciata* and *L. schistorhyncha*) in Philippines, Taiwan and Niue islands [18], [29]–[30] (M. Guinea pers. com). Apart from this important, albeit anecdotal information, oviposition sites remain undiscovered for the six other species of the *Laticauda* genus, and thus for almost all populations of sea kraits. In addition, there are currently no data concerning the ecology of neonates or juveniles.

Two species of sea kraits are still abundant in New Caledonia, the Yellow sea krait (*Laticauda saintgironsi*) and the Blue sea krait (*L. laticaudata*) [31]. Despite long-term studies (>13,000 individuals marked [31]), their reproductive ecology remains poorly known. This lack of information hampers protecting key areas for reproduction [32]. Indeed, populations are disseminated across the immense network of islets spread throughout the lagoon and gravid females may lay their eggs in their home colony. Alternatively, females may converge toward few communal nesting sites as observed in other snake species [33]. Clarifying these alternatives is important in terms of conservation. In the first case, each nesting site must be identified and protected; in the second case, the protection of communal laying site(s) becomes a top priority. In addition, juveniles provide recruits; they are key components of population viability. It is therefore essential to identify and protect their specific habitat(s). This study provides the first ecological data available on juveniles – a very elusive cohort in snakes [34] - and proposes simple practical conservation actions.

## Materials and Methods

### Ethical Note

The procedures were approved by local authorities: Service de la Mer et de la Protection du Lagon, DENV, Province Sud (permits# 6024-179/DRN/ENV, 6024-3601/DRN/ENV and 503/DENV/SMER). Sea snakes, including the endemic *L. saintgironsi*, are not protected or endangered in New Caledonia (Code de l’Environnement de la Province Sud 2009; www.province-sud.nc). New Caledonia regulations are permissive, most of the lagoon, coralline islets, and species are not protected. Thus, we used harmless techniques approved by the far more restrictive French Ethical Committee (COMETHEA, approval # CE2013-5; see [35] for the lack of impact on sea krait populations). Individuals were captured by hand, measured (body size, body mass), palpated, marked (scale clipping; see [31]), and released. No individual was sacrificed or injured.

### Yellow Sea Krait in New Caledonia

This study focuses on the Yellow sea krait, *L. saintgironsi*. This amphibious species is endemic to New Caledonia [36]–[37], and exhibits high crawling and climbing abilities when moving on land [38]–[39]. Large colonies are spread throughout the New Caledonia lagoon (e.g. 22°31′55″N; 166°36′34″E; 22°50′52″N; 166°52′39″E), mostly in small coralline islets but also in the shorelines of the mainland and larger islands [31]. Mark-recapture studies and field experiments demonstrated that individuals exhibit a very high degree of fidelity for their home islet; and more precisely for limited segments of the shore [26], [40].

### Field Procedure

We surveyed 38 sites in the southwest lagoon of New Caledonia. Sampling included remote offshore islets situated near the barrier reef (sometimes >50 km from the coast), and coastal sites (see [31] for a detailed map). During 578 days, we captured 11,356 individuals (8,724 captures and 2,632 recaptures of previously marked individuals). Most snakes (95%) were sexed, measured (snout vent length SVL, to the nearest 0.5 cm, and body mass BM to the nearest g), palpated to assess feeding and reproductive status [41]–[42], and individually marked [31]. Follicles close to maturity are large in sea snakes (sea snakes produce few very large offspring); consequently, palpation provided reliable estimates of clutch size. Some individuals were forced to regurgitate their prey for dietary analyses [41], [43]–[44].

Our long term recapture data enabled us to assign individuals to different age/maturity classes using SVL. During field sessions, snakes smaller than 50 cm SVL were new individuals (not previously captured), and thus they were presumably young-of-the-year because less than a year elapsed between two subsequent field sessions. On many occasions, these small snakes exhibited a typical umbilical scar suggesting that they recently hatched. Recaptures provided further precision. Small sea kraits grow rapidly and thus they can exceed 50 cm SVL in few months (e.g. 3 months, corresponding to the duration of several field sessions). Our recapture data also indicate that during one year, most snakes initially smaller than 50 cm in SVL reach a maximal body size of 65 cm SVL. Consequently, snakes larger than 50 cm SVL, but smaller than 65 cm SVL, were estimated to be older than 3 months and younger than 12 months. Thus, we also considered that snakes smaller than 50 cm SVL were younger than 3 months. For simplicity these very small snakes were referred as neonates. We acknowledge that the precision of these estimates was coarse (± few months); however such imprecision was not likely to alter our main results (see result section).

Males smaller than 63 cm SVL were never observed mating, whereas enlarged follicles were never palpated in females smaller than 75 cm SVL. Therefore, we considered that snakes with a body size ranging from 50 cm SVL to 63 cm SVL in males, or 75 cm SVL in females, were juveniles [43]. Larger snakes were considered as adults. Sexual maturity is not strictly determined at a precise body size; thus, the 60–65 cm SVL size class likely contained both immature and mature males. Similarly, the sexual maturity status of females measuring approximately 75 cm SVL could not be determined with certainty.

Bite-scars caused by prey when they retaliate during capture accumulate over time on the skin of sea kraits [45]. Both neonates and juveniles exhibited very rarely these typical scars. Overall convergent information (e.g. capture/recaptures, palpations, scar occurrence) suggested that our age/maturity assignment based on SVL, although subjected to imprecision around the body size limits (i.e. 50 cm, 65 cm, and 75 cm SVL), was robust. The current study essentially focuses on very small snakes (<50 cm SVL, neonates). Excluding recaptures (few neonates were recaptured, especially during a given field session) from analyses did not change the main outcomes; therefore for simplicity most results presented were based on the total number of observations. Growth rate was calculated as the increase of size between captures.

## Results

### Population Surveys

**Figure 1:**
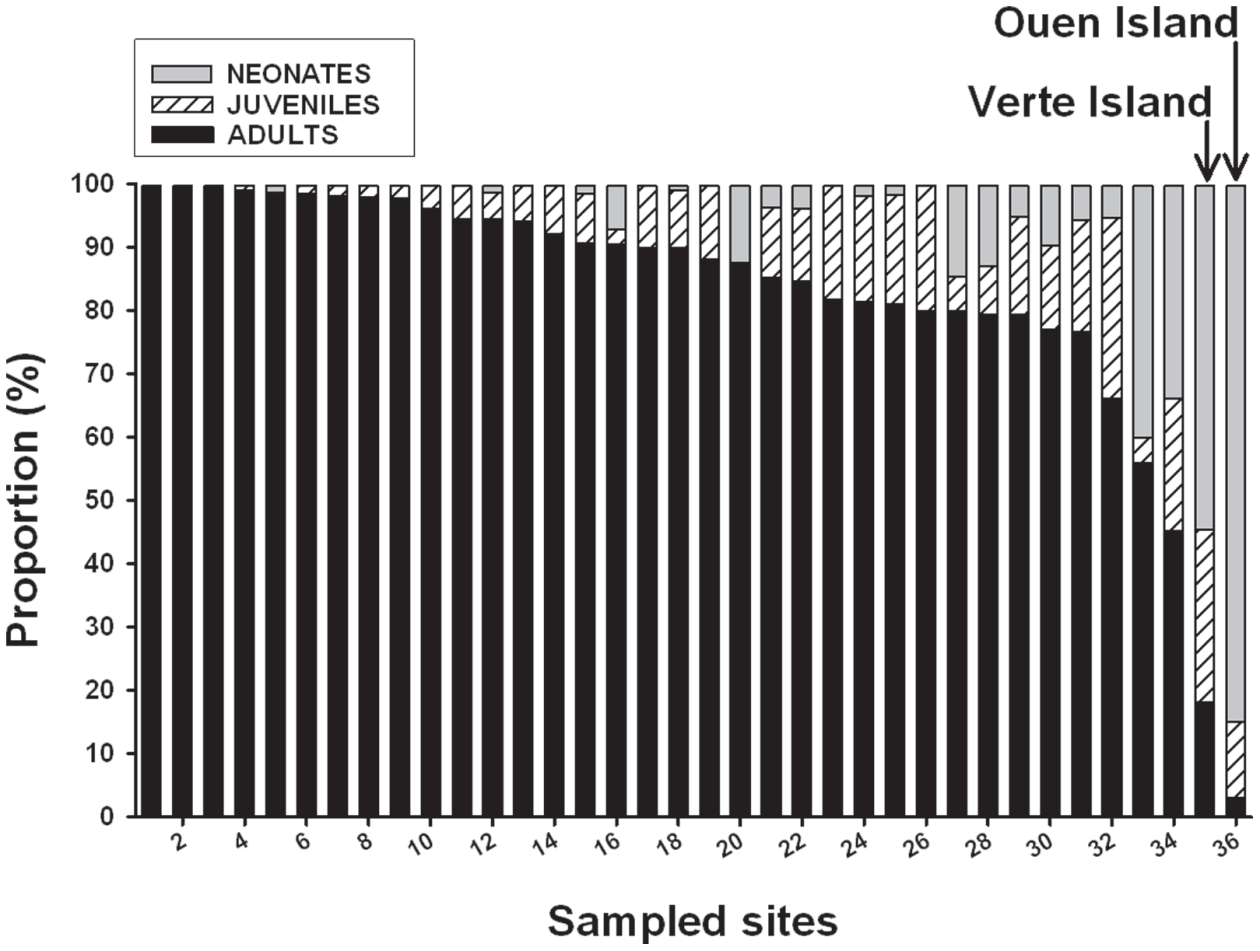
Proportion of neonate (grey), juvenile (hatched) and adult (black bars) sea kraits observed in different populations of New Caledonia (N = 36 sites where the number of snakes exceeded 10 [Mean = 313 snakes per site, total N = 11,273 observations]). The populations have been ranked according to the proportion of adults. The first 20 sites are represented by small flat sandy coralline islets while the last four sites have an igneous or rocky substrate and are situated near, or on the shore of the mainland, see [31] for map details.

In the course of the study we observed a large number of neonates (snakes smaller than 50 cm SVL, N = 971); however relative to the total number of observations, this value represented a small proportion: 8.6%. Importantly, this proportion varied greatly across colonies (Figure 1), ranging from 0.0% (e.g. Améré islet, N = 1,785 snakes) to 84.7% (e.g. Ouen Island, N = 679 snakes). Most snakes smaller than 50 cm were captured at Ouen Island (the nursery, see below). Disregarding this specific site, recalculation provided a lower proportion of neonates: 3.5% (N = 396).

**Figure 2:**
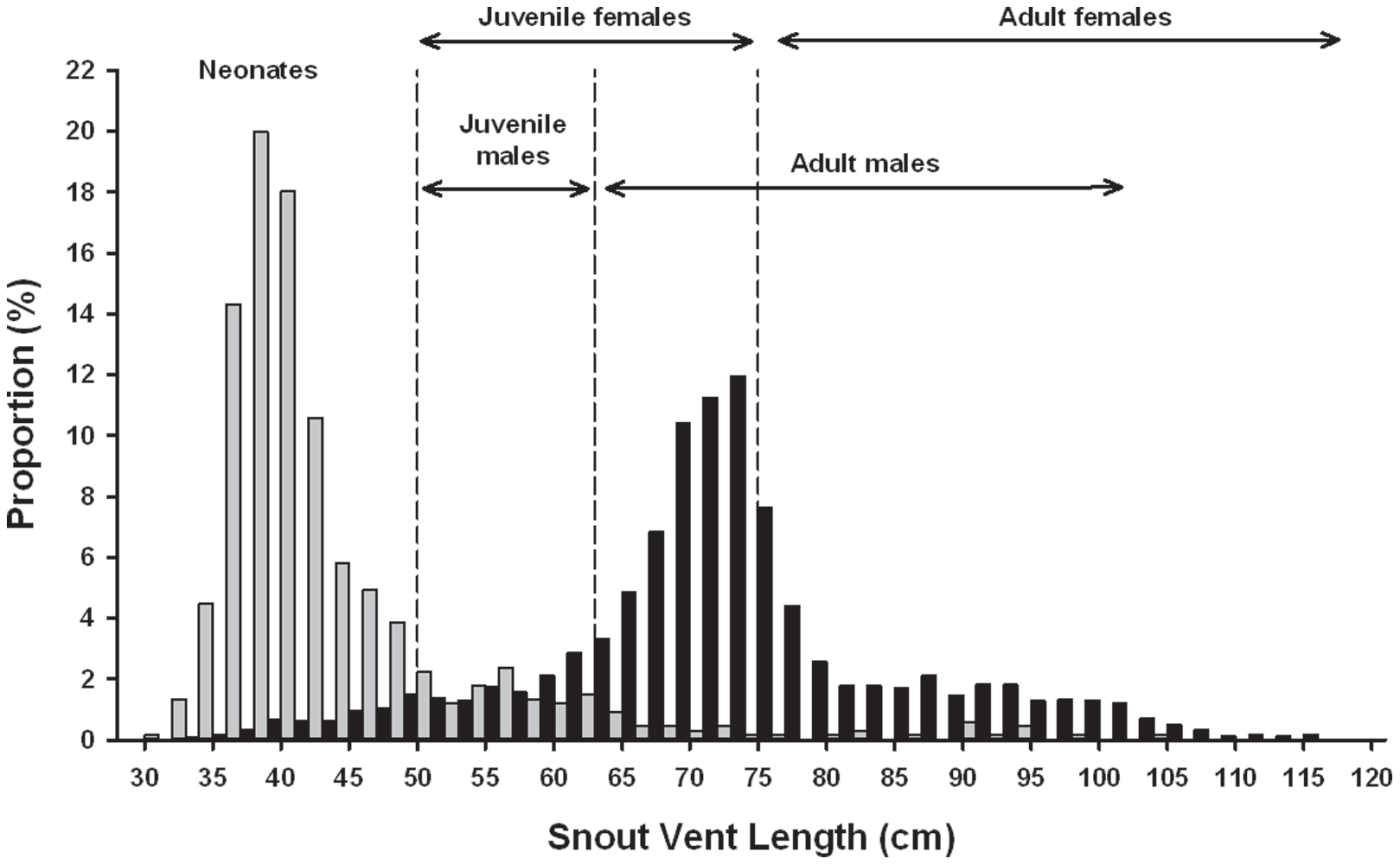
Comparison of the body size distribution (hence age structure) of two islets. The proportion of individuals (Y-axis) in each 2 cm -SVL interval (X-axis) is indicated with gray bars for Ouen Island *versus* black bars for Signal Island. Vertical dashed lines indicate the limit between the main age classes (see text for details). Ouen Island contains a main cohort of neonates, a second smaller cohort of juveniles, and few adults. In strong contrast, Signal Islet contains essentially adults. Both sites have been monitored at least 5 times and they shelter large number of individuals (N = 679 observations in Ouen Island, N = 1,987 in Signal).

In July 2010, XB discovered a peculiar population on the shore of one large island situated near the mainland coast: Ouen Island (22°22′36″S, 166°46′43″E). To our knowledge, this population markedly differ from any other snake populations (either considering marine or terrestrial species), being almost entirely composed of very young snakes (N = 575 neonates, 84.7% of the population; Figure 2). Juveniles larger than 50 cm (SVL, 3–12 months old on average) were also abundant (N = 78, 11.5%); we found only 27 adults (4.0%). Most of the snakes were found deeply sheltered into a narrow thick herbaceous area (1 m×30 m) that covers a 90 m long sea wall. Such a high concentration of small snakes was never observed previously. The young snakes found in this location represented 59.3% of the total number of neonates we observed in New Caledonia.

**Figure 3:**
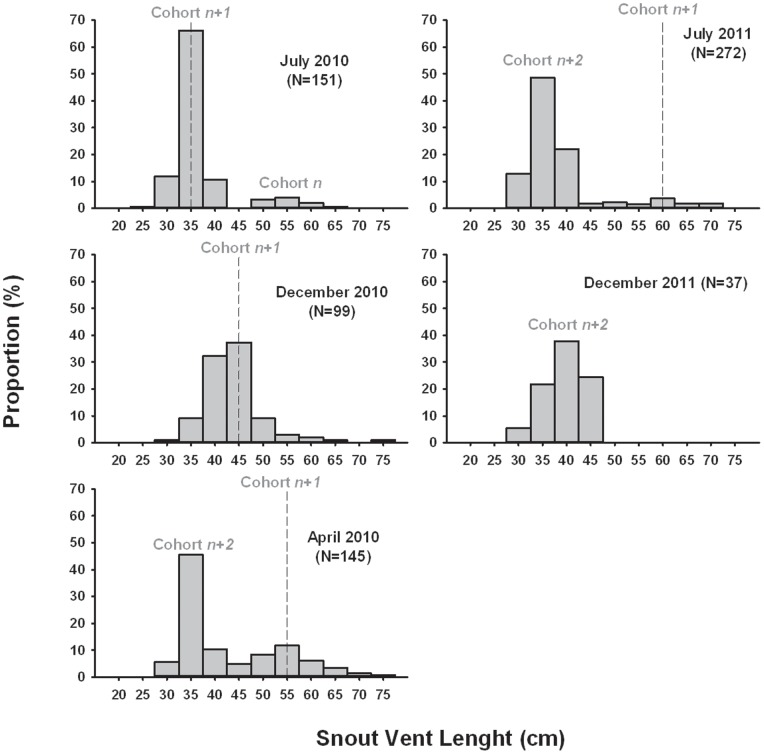
Changes in body size distribution (X-axis) observed in small sea kraits (neonates+juveniles) over time in Ouen Island. The site was monitored during seven sessions (2010–2011), but we pooled sessions that were separated by less than three weeks (e.g. July & August 2011), resulting into 5 main periods. Different size cohorts, hence age cohorts, are visible. Recaptures of several individuals provide a strong support for the suggested succession of cohorts (cohort *n, n+1, n+2*; see text). Neonates (less than 50 cm SVL) grow rapidly and generate a shift toward larger body size from winter (July) to summer (December). This is indicated for the cohort *n+1* with the dashed lines.

We visited Ouen Island on seven occasions at different periods of the year (July 2010 to December 2012), and each time the vast majority of the snakes captured belonged to the neonate and juvenile categories (Figure 3). Therefore, we considered that this location is a nursery for sea kraits.

### Population Structure and Population Size of the Nursery

Disregarding adults, successive size cohorts composed by neonates and larger (older) juveniles were visible at any given period (Figure 3). The neonates were particularly well represented at the end of summer and at the beginning of winter (May – July). The proportion of neonates decreased from early winter to summer while the cohort of larger juveniles increased (Figure 3). This pattern suggests that hatching occurred in late summer to early winter, and that many neonates remained on the nursery sites during several months, some individuals remaining during approximately one year. Recaptures support this pattern: 8 juveniles (5 males, 3 females) marked and recaptured in winter in the nursery markedly increased in size until late spring: growth rate was 0.8 mm day^−1^ (SD = 0.2) in males and 1.1 mm day^−1^ (SD = 0.1) in females.

Crude population size estimates (Schnabel Method, derived from the Lincoln Petersen index and using recaptures between December 2010 and April 2011) yielded a population size of 1,714 individuals (SE = 532). Such estimate was biased because the population was not closed during this period: indeed an unknown proportion of individuals from each age cohort progressively left the nursery, whereas individuals from novel hatchling cohorts were incorporated. Further, during approximately 30 minutes of searching, 50 to 150 small juveniles could be usually captured but these numbers underestimated the actual total number of snakes. Indeed, roughly 50% of the snakes are foraging at sea at any given time [41] and we could not extract all the individuals sheltered into deep refuges (crevices). It is also very unlikely that all the small juveniles of the nursery aggregated in a single location. In fact, we found small juveniles in other places (e.g. under stones in a 200 m radius) whereas very large rocks could not be inspected. Overall, we can confidently estimate that several hundred (possibly thousand) of young snakes were present at the nursery.

### Feeding Rate and Diet

Palpation revealed that 47% (N = 650) of the neonates and juveniles of the nursery had prey in the stomach while, on average, 36% of adult snakes palpated (N = 7,543) had food in the stomach [41]. These two proportions were significantly different χ^2^ = 33.1, df = 1, P<0.001). The main preys were represented by two species of moray eels: *Gymnothorax undulatus* (62%, N = 80) and *G. fimbriatus* (24%, N = 31).

## Discussion

Our study revealed that in New Caledonia, the maintenance of most yellow sea krait populations that are spread over an extremely large area (the lagoon covers ∼24,000 km^2^) actually depends on few coastal nurseries. Indeed, the vast majority of populations contain no, or very small numbers of neonates or juveniles, thus local recruitment is extremely low. Careful long-term surveys of tens of islets in the southwestern-lagoon [31] enabled us to find only two important and highly localized nurseries, respectively situated in two islands separated by 161 km: Verte Islet in the North and Ouen Island in the South (Figure 1). Ouen Island was remarkable, however, for two reasons: first the very high number of neonates and juveniles associated with very small number of adults was very unusual; second, this island is surrounded by many very different colonies. Ouen Island is indeed situated in a very large part of the lagoon; the barrier reef is more than 60 km offshore. This region of the lagoon contains many islets, essentially or exclusively populated by adults, spread from the line-coast to the barrier reef [31]. This pattern suggests that likely hundred females, sometimes from remote islets, converge every year to Ouen Island to lay their eggs.

Sea kraits are characterized by a low fecundity [30]. In *L. saintgironsi*, the mean clutch size is 3.3 [46] and females breed every two years on average (unpublished). Considering the crude neonate number we estimated (∼1,700), more than 500 females laid their eggs in the nursery during the winter 2010. There is no colony that shelters such a corresponding high number of reproductive females (∼1,000 sexually mature females). Adult sea kraits display a very high fidelity for their colony [40] but very few adult females have been observed in Ouen Island. Thus, most of the mothers travelled from distant sites (sometimes more than 50 km) toward Ouen Island, and later returned home after having laid three eggs on average. Ouen Island and possibly other similar nursery locations (yet undiscovered) likely supply a large proportion of juveniles for most populations of yellow sea kraits.

Although we did not find the nests (probably deeply hidden into crevices) and hence could not measure the specific environmental conditions in the laying sites, peculiar environmental conditions that prevail in coastal versus offshore sites might explain why many gravid females converged to the nurseries. In oviparous reptiles, incubation requires well-buffered thermal and hydric conditions [47]–[50]. Ouen Island is a large basaltic island, physically extremely different from the flat sandy coralline islets that shelter most colonies. Coastal sites catch greater amount of rain and ambient temperature is more stable compared to remote islets (http://www.meteo.nc). Thus, rocky coastal shores may provide thermally buffered and relatively humid microhabitats [51] that are suitable for incubation. Interestingly, in the Ouen Island nursery, during the laying period in late November [46], we found 7 adult sea kraits sheltered in crevices under large rocks situated 50 m inshore, 6 were females and 5 were gravid (none was marked). In strong contrast, in offshore sandy islets, large protective rocky structures are lacking, and neonates are lacking too.

The Ouen Island nursery plays another essential role. The clearly multimodal distribution of body sizes that shifted over seasons (and our recapture data) suggests the existence of successive cohorts of young snakes. Many juveniles belonging to each cohort remained in the nursery over prolonged periods (i.e. several months), during which they fed and increased in size. This suggests that many juveniles utilize the nursery before dispersal across the lagoon. Although mortality accounted for an unknown proportion of the progressive decrease in the size of each cohort, dispersal was important in this process as revealed by the very low local recruitment rate and the very small number of adults. Ouen Island nursery functions as dispersal springboard for juveniles. In 2012, we recaptured on an islet situated 18 km away an adult yellow sea krait marked as a neonate in Ouen Island in 2010. This information is anecdotal, but this nonetheless demonstrates the great dispersal ability of very small sea kraits, and the fast growth rate of juveniles.

Overall, protecting nursery locations is a conservation priority to protect laying females, nesting sites, neonates, and also juveniles during a prolonged pre-dispersal phase. In addition, building artificial laying sites in coralline islets may represent an option to promote recruitment in threatened populations; such constructions also offer appropriate shelters to adult snakes [13], [52]–[53]).
